# MAC: Merging Assemblies by Using Adjacency Algebraic Model and Classification

**DOI:** 10.3389/fgene.2019.01396

**Published:** 2020-01-31

**Authors:** Li Tang, Min Li, Fang-Xiang Wu, Yi Pan, Jianxin Wang

**Affiliations:** ^1^School of Computer Science and Engineering, Central South University, Changsha, China; ^2^Division of Biomedical Engineering, University of Saskatchewan, Saskatoon, SK, Canada; ^3^Department of Computer Science, Georgia State University, Atlanta, GA, United States

**Keywords:** adjacency algebraic model, contig classification, contig reconciliation, de novo assembly, next-generation sequencing

## Abstract

With the generation of a large amount of sequencing data, different assemblers have emerged to perform de novo genome assembly. As a single strategy is hard to fit various biases of datasets, none of these tools outperforms the others on all species. The process of assembly reconciliation is to merge multiple assemblies and generate a high-quality consensus assembly. Several assembly reconciliation tools have been proposed. However, the existing reconciliation tools cannot produce a merged assembly which has better contiguity and contains less errors simultaneously, and the results of these tools usually depend on the ranking of input assemblies. In this study, we propose a novel assembly reconciliation tool MAC, which merges assemblies by using the adjacency algebraic model and classification. In order to solve the problem of uneven sequencing depth and sequencing errors, MAC identifies consensus blocks between contig sets to construct an adjacency graph. To solve the problem of repetitive region, MAC employs classification to optimize the adjacency algebraic model. What’s more, MAC designs an overall scoring function to solve the problem of unknown ranking of input assembly sets. The experimental results from four species of GAGE-B demonstrate that MAC outperforms other assembly reconciliation tools.

## Introduction

Next-generation sequencing technologies (NGS) offer a large volume of short sequences with relatively short insert size compared to the traditional Sanger sequencing technology and the third generation sequencing technologies, e.g., Pacific Biosciences (Eid et al., 2009) and Oxford Nanopore (Clarke et al., 2009). Although considerable third generation sequencing data has been produced, due to the higher cost per base and higher sequencing errors, NGS sequencing data still plays an important role in tackling an increasing list of biological problems. The de novo genome assembly is a fundamental process for computational biology (Schatz et al., 2010), which drives the generation of many assemblers to complete the construction of genome sequences, such as Velvet (Zerbino and Birney, 2008), ABySS (Simpson et al., 2009), ALLPATHS- LG (Gnerre and Jaffe, 2011), SOAPdenovo (Li et al., 2010), EPGA2(Luo et al., 2015), Miniasm (Li, 2015), BOSS (Luo et al., 2017), SCOP (Li et al., 2018a), ARC (Liao et al., 2018), iLSLS (Li et al., 2018b), MEC (Wu et al., 2017), EPGA-SC (Liao et al., 2019a), PE-Trimmer (Liao et al., 2019b), and so on.

However, there is no single assembler that could perform optimally in every quality metric, which has been demonstrated repeatedly (Earl et al., 2011; Salzberg et al., 2012; Bradnam et al., 2013). The situation is caused by various factors: (1) Assembly algorithms are mainly based on overlap-layout-consensus graphs or de Bruijn graphs, these two types of algorithms use different strategies to deal with errors, inconsistencies, and ambiguities; (2) NGS genome assemblies suffer from long repeats and duplications, which is the primary reason why some assemblers outperform others in specific regions and specific species (Alkan et al., 2010); (3) the uneven sequencing coverage of NGS data increases the complexity of assembly, which makes the parameters having great influence on the assembly results, such as k-mer size; (4) the sequencing errors and chimeric reads cause direct assembly mistakes. Besides, different sequencing platforms usually introduce different bias (Harismendy et al., 2009), so the assemblies generated by various platforms may present different features, and there is usually complementarity between them (Diguistini et al., 2009). Thus, it is appealing merging different assemblies to generate a high-quality assembly by using complementary, which is first proposed by Zimin et al., called assembly reconciliation. The main goal of assembly reconciliation is to increase the contiguity of assembly results while reducing (or at least not increasing) the errors in assembly.

Many assembly reconciliation algorithms have been proposed, for some earlier ones, such as Reconciliator (Zimin et al., 2005) and GAM (Casagrande et al., 2009). Reconciliator detects apparent errors in the assembly, and then the error regions are modified by using the alternative draft assembly, through which the gaps between sequences are reduced. GAM defined supercontig to facilitate the integration, which takes two assemblies as input, and regards the former one as reference. For some reference-based algorithms, such as eRGA (Francesco et al., 2011), RAGOUT (Kolmogorov et al., 2014), and MAIA (Nijkamp et al., 2010), if there is no corresponding reference or relative reference genome, they cannot work properly, so we don’t discuss these methods here. The algorithm CISA is used to integrate the assemblies of bacterial genome in the four major phases (Lin and Liao, 2013). Firstly, CISA extracts the largest contig as a representative contig, and aligns the remaining contigs to the representative coting, then conducts extension with the contig whose alignment rate is more than 80%. This step is repeated iteratively until there is no representative contig found. Secondly, CISA identifies two types of misassemble contigs: for the misjoined error, CISA removes the contig; for the insertion error, CISA splits the contig. Thirdly, CISA merges contigs which have at least 30% overlap, and also estimates the size of repeats. Finally, if the overlap between two contigs is greater than the maximum size of repeats, CISA merges the contigs. CISA could be used to merge more than two assemblies.

The objective of GAA is to generate an accordance assembly from two or more large genome assemblies (Yao et al., 2012). GAA takes a target assembly and a query assembly as input, then uses BLAT aligner (Kent, 2002) to align the query assembly to target assembly. The high scoring matches are used to construct the accordance graph, GAA finds the maximal sub-paths from the graph, and the gaps can be divided into two types, between contigs and inside contigs. For the gaps between contigs, GAA compares the observed value and expected value of gap size, then decides whether to merge two contigs. For the gaps inside contigs, a compression-expansion(CE) statistic module (Zimin et al., 2005) is used to evaluate the gap regions. The 454 and Illumina de novo assemblies are used to examine the performance of GAA.

GAM-NGS (Vicedomini et al., 2013) is the updated version of GAM, GAM-NGS can be used on all NGS-based assemblies, especially for eukaryote genomes. Two assemblies and a SAM alignment file are taken as input, GAM-NGS first searches the mapping file to identify highly similar fragments between two assemblies, which is called “blocks”, then a graph is used to record and weight the information of blocks, and the conflicts are resolved in the graph. A semi-global alignment between contigs is computed by GAM-NGS, and two contigs are merged if the identity between them is larger than 95%. The CE statistic module (Zimin et al., 2005) is used to choose which assembly can be merged.

The main purpose of MIX (Soueidan et al., 2013) is to reduce both the fragmentation of contig sets and reduce the time consumption of genome finishing. MIX builds an extension graph where vertices represent the terminals of contigs, and the edges represent the alignment situation between contigs. MIX attempts to solve the maximal independent longest path set, which is NP-hard. The performance of algorithm is evaluated on the GAGE-B (Tanja et al., 2013) bacterial dataset.

Metassembly (Wences and Schatz, 2015) merges all the input assemblies into a final one, which is better than or as good as the original assemblies. Metassembly regards one of the inputs as a “primary” assembly, then the others are “secondary” assemblies, the secondary assemblies are used to add useful information to the primary assembly. A pairwise algorithm is used to merge multiple assemblies, the primary assembly is aligned to the secondary assembly, and the best aligned position can be evaluated by LIS (longest increasing subsequence) function. The CE statistic (Zimin et al., 2005) is used to assess the conflicts and select the locally best sequence.

In general, most of the methods described above are based on the CE statistic (Zimin et al., 2005), which is used to detect compression or expansion misassemblies between two input assemblies. However, the CE statistic is obtained by aligning paired-end or mate-pair reads to the assembly, which is impacted by the alignment quality and the false positive within error detection leads to the misassembly directly. In addition, most of the current reconciliation tools are designed for merging short sequences (<100bp), like CISA and GAM-NGS, which performed poorly in merging longer sequences (>200bp). Therefore, there is an urgent require for the robust reconciliation tool to increase the length and quality of assembly, as well as adapt to longer sequencing data.

In this study, we propose a novel assembly reconciliation tool, named MAC, which uses alignment information and GC-content of paired-end reads to classify all the contigs into two types. Then, consensus blocks between contig sets are identified, and the unreliable fragments caused by uneven sequencing depth or sequencing errors could be filtered out. In addition, MAC utilizes the adjacency algebraic model to facilitate the merging process, in which the adjacent graph is used to fulfill accurate fusions between consensus blocks. The classification result of contigs is used to optimize the model, and the repetitive regions could be eliminated by splitting contigs and reconstructing the adjacent graph. What’s more, an overall scoring function is proposed to solve the problem of unknown ranking of input assemblies, the scoring function evaluates the overall quality of assembly sets by alignment quality and coverage information. The experimental results from the datasets of GAGE-B demonstrate that MAC performs better than other reconciliation tools.

## Method

**Figure 1:**
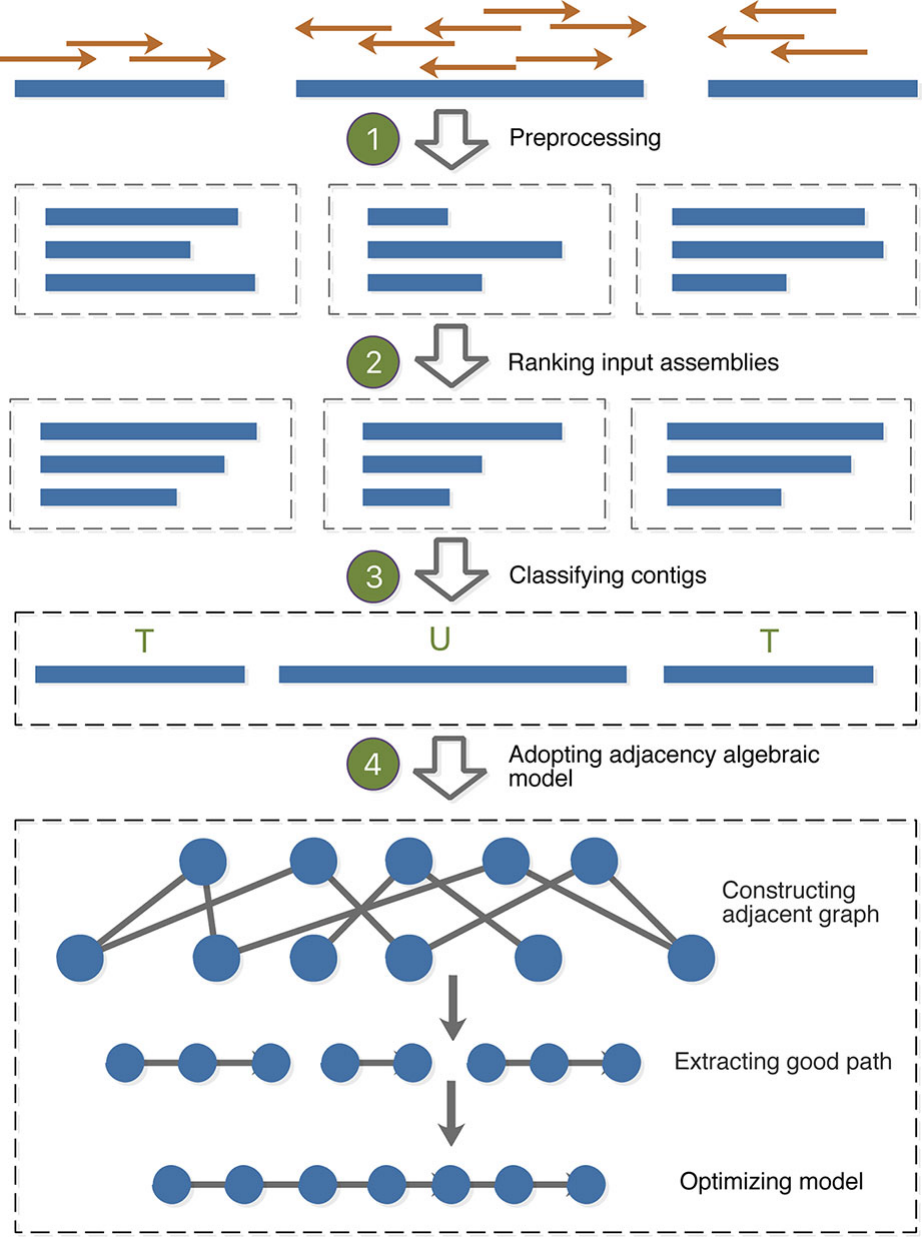
Flowchart of MAC algorithm.

MAC employs the adjacency algebraic model (Sankoff et al., 2000) and the classification to merge assemblies. The identification of consensus blocks is to filter out the unreliable fragments caused by uneven sequencing depth and sequencing errors; the addition of classification is to optimize the adjacency algebraic model and eliminate the influence of repetitive regions. The outline for the whole algorithm is as follows: (1) Preprocessing: MAC aligns paired-end/mate-pair reads to contig sets, and filters out the low-quality alignment; (2) Ranking input assemblies: MAC designs an overall scoring function to rank the input assemblies; (3) Classifying contigs: MAC utilizes the alignment results and GC-content of paired reads to classify contigs; (4) Adopting the adjacent algebraic model: MAC constructs an adjacent graph to fulfill some accurate fusions of consensus blocks, then uses classification results of contigs to optimize the remaining processing steps. The flowchart of MAC algorithm is shown in **Figure 1**.

### Preprocessing

MAC takes multiple contig sets and paired-end/mate-pair reads as input, the aligner Bowtie2 needs to be installed in advance. The input reads are aligned to each contig set, respectively. For reads aligning to multiple positions, MAC only maintains the highest score alignment for each read, and removes the redundant alignments. According to the paper of Luo et al., the length of insert size follows a normal distribution N (*μ_is_*, *σ_is_*), so the distance between two paired reads, which align to the same contig, should be in the range of [*μ_is_*– 3*σ_is_, μ_is_+ 3*σ_is_]. For the reads which violate this rule, MAC removes the corresponding alignment. To reduce the impact of sequencing errors, MAC extracts the sequencing quality of every base in reads, and calculates the average and standard deviation of sequencing quality for the remaining alignment, denoted by M_q_ and *σ_ra_*, respectively. Let *Q_i_* represent the mean value of sequencing quality for the *i-*th alignment. If *Q_i_* < M_q_- 3*σ_ra_, MAC removes the alignment information.

### Ranking Input Assemblies

As most existing assembly reconciliation tools depend on the ranking of input, and the results usually change when the order of input assemblies change. To achieve better results, users have to evaluate the contiguity and correctness of every input assembly by taking the reference into Quast (Gurevich et al., 2013) or other evaluation tools. In the study, MAC utilizes the mapping quality and read coverage to rank the input assemblies. The compact idiosyncratic gapped alignment report (CIGAR) can be obtained from files in the SAM format, in which “M” represents match/mismatch, “I” represents insertion, “D” represents deletion, and the number before a character represents its corresponding quantity. Assume that the length of contig *C* is *L*, *j (1≤j≤L)* is the position at *C*, *q_j_* represents the CIGAR of position *j*, which is calculated as follows.

(1) $$q_{j}=\{\begin{array}{l} 1,\;\text{if}\;j=M\\ -1,\;\text{if}\;j=M^{*}\mathrm{or}\;I\;\mathrm{or}\;D, \end{array}$$

where M denotes match and M^*^ denotes mismatch. In fact, we cannot distinguish match and mismatch from a single character “M”, so MAC calculates the average mapping score of the SAM file. If the mapping score of the corresponding read is larger than or equal to the average mapping score, the character “M” is thought to be match, otherwise, “M” is thought to be mismatch.

To take the coverage into consideration, MAC extracts the alignment of contig *C*, to calculate the average *rc*, and standard deviation *σ_rc_* of read coverage. Assume that *rc_i_* is the read coverage of the spanning region of read *i*, *RC* is used to indicate whether the coverage of the region deviates too much, which is computed as follows.

(2) $$RC=\{\begin{array}{l} 1,\;\mathrm{if}\;({\mathrm{rc}}_{i}>\mathrm{rc}+2*\sigma_{\mathrm{rc}})\;\mathrm{or}\;({\mathrm{rc}}_{i}<\mathrm{rc}-2*\sigma_{\mathrm{rc}})\;\\ -1,\;\mathrm{otherwise} \end{array}$$

In order to comprehensively consider the mapping quality and read coverage, MAC employs an overall scoring function to rank the input assemblies, which is calculated as follows.

(3) $$\mathrm{score}=\frac{\sum_{c=1}^{N}\sum_{j=1}^{L}q_{j}-\sum_{c=1}^{N}\sum_{i=1}^{K}\mathrm{RC}}{N}$$

### Classifying Contigs

In this step, MAC evaluates the quality of contigs by using the alignment result and GC-content, and then classifies all the contigs into two types. Due to the problems of sequencing errors, uneven depth, existence of repetitive regions and the bias of algorithm strategy, contigs often contain misassemblies, which influence the subsequent assemblies directly. Therefore, MAC estimates the correctness of contigs, and marks the type for every contig, and records the potential error positions.

For a contig *C*, whose length is *L*, the coordinate of position *j* is in the range of [1, *L*]. The fragment coverage *fc(j)* could be defined as the number of reads with the high alignment scores which span the position *j*. Because low coverage regions more likely contain error joints, MAC employs a cutoff fc* to identify the potential error positions, fc* can be calculated by the average of fragment coverage for all the positions of contig C as follows (Wu et al., 2017).

(4) $${\mathrm{fc}}^{*}=\alpha *\frac{\sum_{j=1}^{L}\mathrm{fc}(j)}{L}$$

The parameter *α* can be set by users. If the fragment coverage of position *j* is less than the cutoff, that is fc(j) ≤ fc*, the position *j* is regarded as a potential error position. If there are multiple continuous potential error positions, the region covering these positions can be group into a region set *T* (*T = {[m, n]* | n ≥ m, ∀ j∈ [m, n], fc(j) ≤ fc*}). For every region in set *T*, MAC chooses the position whose fragment coverage is the lowest as the breakpoint, *B_p_* (*m≤ B_p_≤ n*).

Owing to the uneven sequencing depth, some low-depth regions may be mistakenly categorized as containing error positions. Therefore, MAC estimates the coverage condition of the neighbor flanking regions for breakpoint *B_p_* to reduce false positives. *M_s_* is the number of paired reads whose left mate maps to the left flanking region of *B_p_*, and right mate maps to other contigs. *M_p_* is the number of paired reads whose right mate maps to the right flanking region of *B_p_*, and left mate maps to other contigs. Then two rates: *P_cs_* and *P_cp_*, are calculated as follows (Wu et al., 2017).

(5) $$P_{\mathrm{cs}}=\frac{fc(B_{p})}{fc(B_{p})+M_{s}}$$

(6) $$P_{cp}=\frac{fc(B_{p})}{fc(B_{p})+M_{p}}$$

*P_cs_* and *P_cp_* are used to estimate whether the region [m, n] is low-depth or not. If *P_cs_>β* or *P_cp_>β*, the region is thought to be a low-depth region, and should be removed from the potential set.

Owing to the GC-content bias, some regions may cover less reads or even no reads, and these regions are mistakenly categorized as containing error positions. Therefore, MAC evaluates whether the GC-content of the neighbor flanking regions for *B_p_* is too high or too low. *P_GC_* is the GC-content rate of the potential error region which contains *B_p_*, and *P_g_* is the GC-content of the whole genome, *P_g_* is calculated as follows (Wu et al., 2017).

(7) $$P_{g}=\frac{\sum_{i=1}^{N}\sum_{j=1}^{L_{i}}I_{j}}{\sum_{i=1}^{N}L_{i}},$$

where N represents the number of contigs, *L_i_* is the length of the *i*-th contig, *I_j_* is an indicator variable, when the base at position *i* is G or C, *I_j_* equals to 1, otherwise, *I_j_* equals to 0. If *P_GC_* ≥ *P_g_* + 1, the region is thought to be GC-rich, otherwise, the region is thought to be GC-poor. Both GC-rich and GC-poor regions are removed from the potential set.

After removing the low-depth regions and GC-bias regions, the remaining single potential positions and potential regions are certainly false. which satisfy the following conditions at the same time:

① *fc(j)* ≤ fc*;
② *P_cs_* ≤ *β* and *P_cp_* ≤ *β*;
③ *P_GC_* < *P_g_* + 1 and *P_GC_* > *P_g_* + 1.

The regions estimated as low-depth or GC-bias are thought to be uncertain regions, and the positions in these regions satisfy the following conditions simultaneously:

① *fc(j)* ≤ fc*;
② *P_cs_* > *β* or *P_cp_* > *β*;
③ *P_GC_* ≥ *P_g_* + 1 or *P_GC_ ≤ P_g_* + 1.

After excluding the above two types of positions, the rest positions are certainly true. For the certainly false positions/regions, MAC breaks the corresponding contigs at the false position or the *B_p_* position of the false region, and eliminate certainly false positions. Based on the above evaluation, all the input contigs can be divided into two types: Uncertain (U) and True (T). If the contig contains one or more uncertain regions, the contig is classified as U contig, while the contig only containing true positions is classified as T contig.

### Adopting Adjacency Algebraic Model

The order of merging is determined by the ranking of overall scores, which are calculated in the previous step. MAC merges two assemblies at a time, the next assembly and the resultant assembly are merged iteratively. In the merging process, MAC utilizes an adjacency algebraic model (Sankoff et al., 2000) to find the conjunctions between contigs. The adjacency algebraic model was introduced by Feijã£O and Meidanis to find a permutation to minimize the algebraic rearrangement distance (Feijã£O and Meidanis, 2013), and the adjacency algebraic model was proved to be efficient on the problem of contig ordering (Chen et al., 2018). In this study, MAC uses the adjacent graph to represent the adjacency algebraic model and utilizes the classification of contigs to optimize the model, the pseudo-code of adopting the adjacency algebraic model is as shown in **Algorithm 1 of Supplementary Material**.

#### Constructing Adjacent Graph

**Figure 2:**
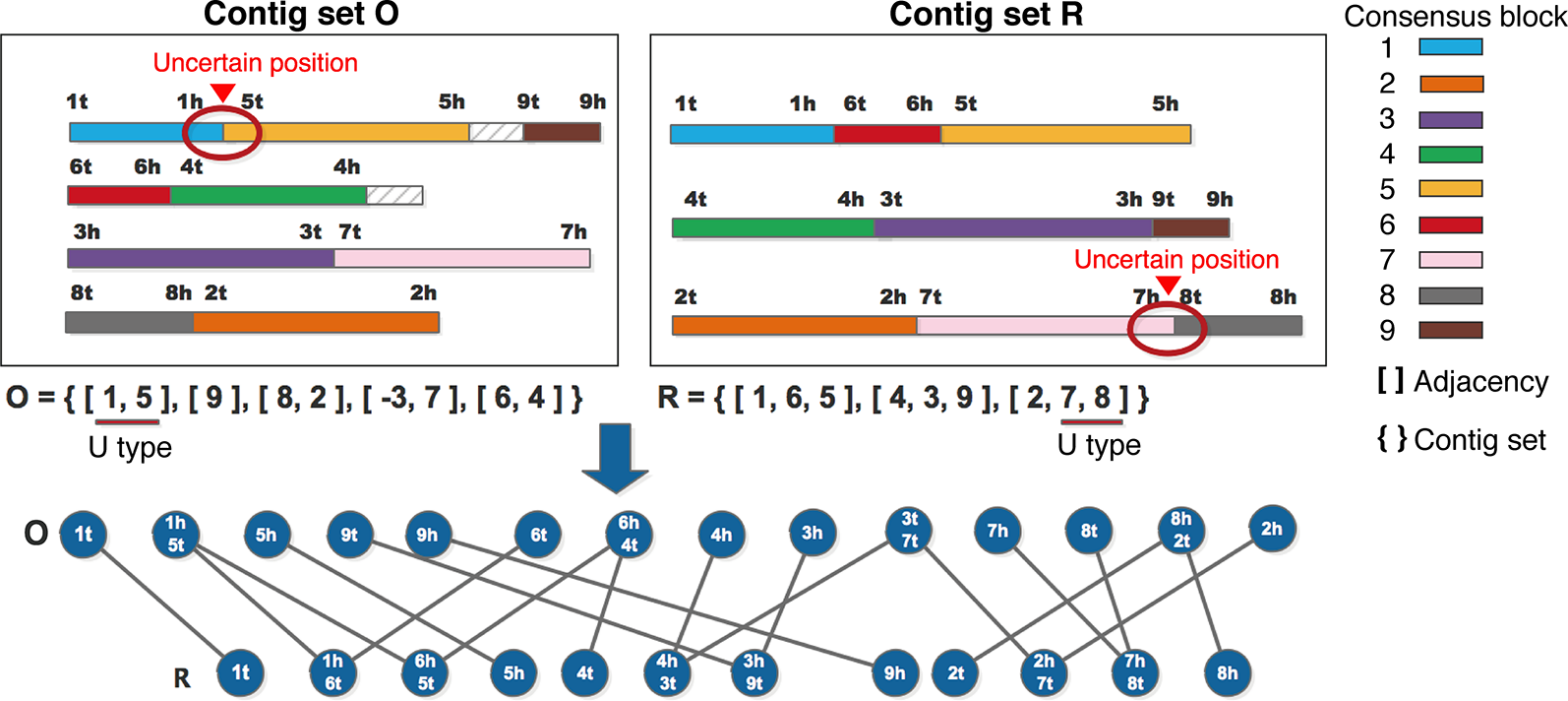
An example for constructing adjacent graph.

Given two input contig sets *O* and *R*, MAC utilizes the NUCmer package from MUMmer (Kurtz et al., 2004) to identify the high similarity fragments between *O* and *R*, which are called “consensus blocks”, and numbers these consensus blocks. Two consensus blocks are thought to be adjacent, if they are next to each other, or if they overlap each other end-to-end with the overlapping length of *l* (l ≤ l_cmin_* 0.01), where *l_cmin_* is the smaller one between the lengths of two consensus blocks, *l* is called the adjacent region. In general, there are two or more consensus blocks in one contig, and the consensus blocks may connect with each other, or maybe contain intervals between them. As described above, every contig can be divided into two types: “U” and “T”. For the “U” type of contigs, if potential error positions locate at the adjacent regions of consensus blocks, the position information is retained. Otherwise, if potential error positions locate at the center region of consensus blocks, these positions are thought to be reliable, and could be removed form the potential error set. For the “T” type of contigs, MAC retains its state. MAC distributes the orientation for every consensus block, and uses tail(“t”) to denote the starting position, head(“h”) to denote the ending position. As shown in the example of **Figure 2**, 9 consensus blocks are found between two contig sets *O* and *R*, the adjacent relationships are enclosed in brackets, so *O* and *R* can be represented by *O* = {[1, 5], [9], [8, 2], [-3, 7], [6, 4]}, *R* = {[1, 6, 5], [4, 3], [2, 7, 8]}, respectively. As the orientation of consensus block “3” in *O* is reversed (from 3h to 3t), we use “-3” to represent this consensus block in *O*. In **Figure 2**, we suppose that there were uncertain positions between [1,5] in the first contig of O and [7,8] in the third contig of R, so these two contigs were regarded as “U” type, which are marked by red cycles on the contigs, and the corresponding consensus blocks are also marked with underlines in **Figure 2**, the detail classification strategy has been described above.

Then the adjacent graph *G* = <*V*, *E* > is constructed, *V* is the vertices set of the adjacent graph, the single terminals or conjunctions of consensus blocks are regarded as vertices, in the example of **Figure 2**, 1t, 9t, 9h, 6t, and so on are the single terminals of *O* set, 1h5t, 6h4t, and so on are the conjunctions of *O* set. *E* is the edges set of the adjacent graph, an edge is added between *O* and *R* if two vertices have a terminal in common, such as 1h5t of *O* and 1h6t of *R* both have 1h, so there is an edge between 1h5t and 1h6t.

#### Extracting Good Paths

The major objective of the adjacent algebraic model is to minimize the algebraic distance between two contig sets, which can be denoted by $d\left(O,R\right)=N-C-\frac{P}{2}$ (Feijã£O and Meidanis, 2013), where *N* represents the number of contigs, *C* represents the number of cycles, *P* represents the number of paths in the adjacent graph *G*.

Through the demonstration of Lu et al., getting the minimum algebraic distance is equivalent to obtaining the maximum number of cycles, and the term of “good path” is defined for the cycle (closing path), which can connect multiple consensus blocks to generate a longer assembly. Here we define the conjunctions between two consensus blocks as adjacency, which are enclosed in square brackets in **Figure 2**. The paths in adjacent graph can be summarized according to the length, whether two ends of the path in the same set or in the same adjacency or not. We list all the possible combinations of the features mentioned above, as shown in **Table 1** there are 9 types of combinations in total. In the adjacent graph, two ends of the path appear in the same contig only if they appear in the same contig set, so if two ends of the path cannot be found in the same contig set, they cannot be found in the same contig or adjacency, thus for the types of No.3 and 7 in **Table 1**, two ends of the paths are in different contig sets, they cannot in the same adjacency, here we use “-” to represent the type is absent. If the length of path is odd, two ends should be found in different contig sets, so types of No.1 and 2 are absent. If the length of path is even, two terminals should be found in the same set, so type of No.8 is absent. However, there is an exceptional case, when two terminals form a circle, they can be found in different sets and different adjacencies.

**Table 1:** Nine types of paths in the adjacent graph.

No.	Length of path	In the same set	In the same adjacency	Type
1	Odd	Y	Y	–
2	Odd	Y	N	–
3	Odd	N	Y	–
4	Odd	N	N	Poor-1
5	Even	Y	Y	Poor-2
6	Even	Y	N	Good
7	Even	N	Y	–
8	Even	N	N	–
9	Even	N	N	Circle

From **Table 1**, four types are absent, and the type of No.6 is regarded as a good path, whose length is even, both of the ends are in the same set but in different adjacencies. There are two kinds of poor paths: No.4 and No.5. As the example in **Figure 2** shows, the paths of {4h, 4h3t, 3t7t, 2h7t, 2h}, {9t, 3h9t, 3h}, {7h, 7h8t, 8t} are good, which can form the cycles of [4h, 2h], [9t, 3h], and [7h, 8t]. Through the fusion of [4h, 2h], adjacencies [8, 2] and [6, 4] can be joined into [8, 2, -4, -6]. Through the fusion of [9t, 3h], adjacencies [9] and [-3, 7] can be joined into [-9, -3, 7]. Through the fusion of [7h, 8t], adjacencies [8, 2] and [-3, 7] can be joined into [-3, 7, 8, 2], and these two newly generated results can be further merged into [-9, -3, 7, 8, 2, -4, -6], equals to [6, 4, -2, -8, -7, 3, 9].

#### Optimizing the Adjacency Algebraic Model

In the study of Lu et al., two odd paths (No.4 in **Table 1**) are chosen to join into a cycle repeatedly, until the odd path graph becomes an alternating cycle with the length of two. The remaining No.4 and No.5 paths can be arbitrarily joined together into two longer paths. However, in the actual implementation process, they found the fusion of these two types of paths resulting in error joints. In this study, MAC utilizes the classification of contigs to optimize the processing of poor paths in the adjacency algebraic model. Due to the circle paths in the graph represent the same adjacencies between two sets, so MAC maintains these paths without any process.

As described above, all the input contigs are divided into two types: Uncertain (“U”) and True (“T”), the classification result is stored in the form of a label for every contig together with the potential error positions. After extracting good paths from the adjacent graph, the poor-1 type of paths can be further divided into two sub-types: single path and non-single path. The length of single path is 1, and two terminals are the same, for example, {1t, 1t}, {5h, 5h} and {3h, 3h} in **Figure 2** are single paths, {6t, 1h6t, 1h5t, 6h5t, 6h4t, 4t} is a non-single path. MAC uses the following steps to process poor paths:

- For non-single paths, MAC extracts the adjacencies which are included in the path, then checks the classification of contigs where the adjacencies are located. If contig is “U”, and there is potential error position locating at adjacent region *l*, MAC splits the contig at the potential error position, and then reconstructs the sub-graph to extract good paths again.
- For single paths, MAC does not take any treatment, because during the process of graph reconstruction, some single paths would be eliminated automatically.
- For poor-2 type of paths, if both terminals of a poor-2 path appears in any good path, then the poor-2 path is thought to be spurious, and MAC removes this path along with the contig contained in the path. Otherwise, the poor-2 path can be retained temporarily.

MAC repeats these processing steps iteratively until there is no good path added, and single paths are merged into good paths to generate new adjacencies. For the new adjacencies, if there are overlapping blocks, the shorter adjacency is merged into a longer one. Here we use the same example as **Figure 2** to explain the optimization process, and the detail procedure is as shown in **Figure 2**.

**Figure 3:**
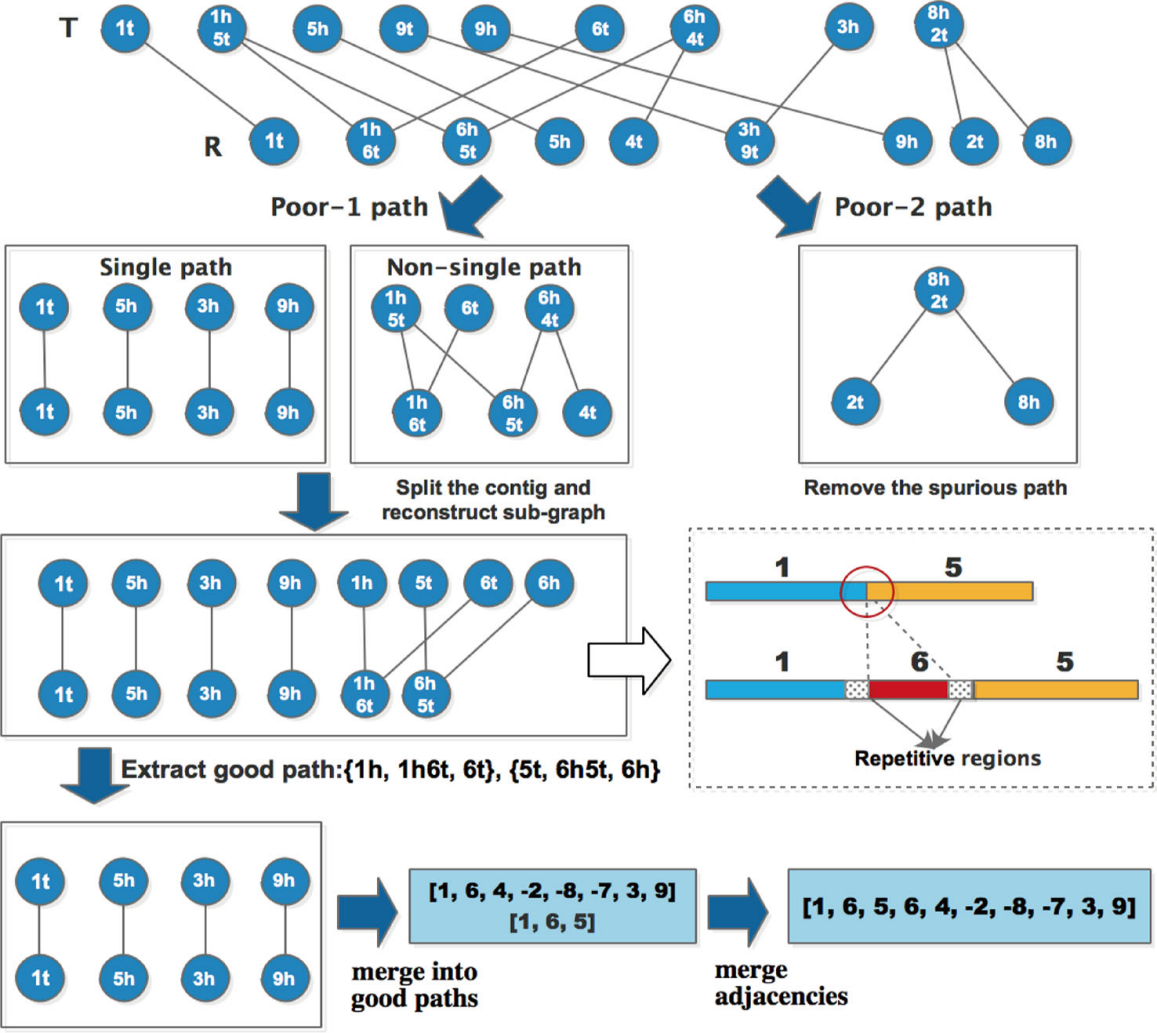
Detail of optimization process.

After extracting good paths for the first time, the remaining are two types of poor paths. For non-single path {6t, 1h6t, 1h5t, 6h5t, 6h4t, 4t}, the adjacency included in the path are: [1, 5], [6, 4] in set *O* and contig [1, 6, 5] in set *R*, according to the processing steps, the error position of adjacency [1, 5] locates at the adjacent region, so the first contig of *O* should be split at the position. In fact, the head of block 1 and the head of block 6 contain the same repetitive sequences, which cause a misjoin between 1 and 5, as shown in the dashed line box of **Figure 3**. As such MAC could solve the problem of repetitive regions. Then the sub-graph is reconstructed, two more good paths are extracted, and there are four single paths remaining. After merging these single paths into good paths, the final contigs can be represented by the adjacencies: [1, 6, 4, -2, -8, -7, 3, 9] and [1, 6, 5]. MAC identifies there is an overlapping region between two adjacencies, and thus merges [1, 6, 5] into [1, 6, 4, -2, -8, -7, 3, 9] to get the final adjacency [1, 6, 5, 6, 4, -2, -8, -7, 3, 9].

## Experimental Results and Discussion

### Datasets and Evaluation Metrics

In this study, we perform the experiments on four real bacterial genomes: M.abscessus, B.fragilis, R.sphaeroides and V.cholerae from GAGE-B (Genome Assembly Gold-standard Evaluation for Bacteria) (Tanja et al., 2013), GAGE-B evaluates the performance of multiple genome assemblers on a spectrum of bacterial genomes sequenced by the sequencing technologies of MiSeq and HiSeq. Here, we use the sequences generated by MiSeq technology, the average read length of these four species is 250 bp, the coverage is 100x, and the genome sizes are 5.1 Mb, 5.4 MB, 4.6 Mb, and 4.0 Mb, respectively. The insert sizes are 335 bp, 600 bp, 540 bp, and 335 bp, respectively. The detail information of raw data is listed in **Table S1** of Supplementary Material. All the assemblies and paired-end reads are available at the website of GAGE-B (http://ccb.jhu.edu/gage_b/).

The evaluation tool Quast (Gurevich et al., 2013) is used to estimate the contiguity and correctness of assemblies. For the metrics provided by Quast, N50 is the metric to evaluate contiguity without reference, and NGA50 could compare the assemblies to a reference genome to get more accurate and comprehensive evaluation. The number of misassemblies is an important metric to measure the correctness of assemblies. In most cases, the increase of N50 and NGA50 inevitably leads to more misassemblies. The contig sets generated by different tools are evaluated in **Tables S2–S5** of Supplementary Material. The major objective of MAC is to increase the contiguity of assemblies, at the same time make sure the number of misassemblies reducing or at least not increasing.

### Experimental Results

Although lots of assembly reconciliation tools have been developed, some of the tools have stopped updating, some need the reference of relative species, and some tools don’t fit for merging relatively longer next-generation sequences which are in ~250bp. Therefore, we exclude these unsuitable tools, and only compare MAC with three assembly reconciliation tools: GAA, MIX and Metassembler. The assemblies are generated by Velvet, ABySS and SOAPdenovo. The contigs generated by Velvet are fragmental and with poor contiguity. ABySS could provide more reliable contigs which have less misassemblies. SOAPdenovo is a powerful tool which produces higher contiguity and correctness contigs. In the experiment, we test the merging performance of four reconciliation tools on the assemblies which have different features. The experiment results are shown in **Tables 2**–**5**. For each dataset, we take the experiments on two assemblies as input (Velvet+ABySS), and multiple assemblies as input (Velvet+ABySS+SOAPdenovo). “MA” in tables represent the numbers of misassemblies.

**Table 2:** The experimental results of *M. abscessus*.

	Contigs num	Largest contig	Size	Genome fraction	N50	NGA50	MA
Velvet	203	226,629	5,136,825	98.965	48,155	41,485	54
ABySS	149	245,660	5,116,522	98.926	70,424	68,549	2
SOAPdenovo	91	286,460	5,133,667	99.139	131,561	113,272	19
*(Velvet+ABySS)*							
GAA	339	129,152	5,152,501	99.094	39,271	37,715	61
MIX	118	245,660	5,376,417	98.891	108,584	70,302	**18**
Metassembler	200	226,629	5,130,215	98.944	48,155	41,485	54
MAC	190	317,945	9,856,881	99.304	**163,219**	**90,766**	58
*(Velvet+ABySS+SOAP)*							
GAA	211	210,497	5,146,833	99.129	54,850	50,904	55
MIX	91	286,460	5,133,667	99.139	131,561	113,272	17
Metassembler	191	226,629	4,934,916	95.03	47,284	39,706	64
MAC	80	287,168	5,146,285	99.249	**141,537**	**131,561**	**10**

The bolded data indicates the highest value of N50 or NGA50 within each comparison.

**Table 3:** The experimental results of *B. fragilis*.

	Contigs num	Largest contig	Size	Genome fraction	N50	NGA50	MA
Velvet	373	91,844	5,310,336	97.661	24,465	24,465	3
ABySS	87	430,487	5,380,960	98.451	130,570	130,570	2
SOAPdenovo	79	606,530	5,341,631	98.226	246,346	246,346	0
*(Velvet+ABySS)*							
GAA	2053	16,951	10,676,299	98.811	4,999	4,999	4
MIX	87	430,487	5,380,960	98.451	130,570	130,570	**2**
Metassembler	256	127,644	5,317,077	97.819	40,339	39,580	3
MAC	136	568,455	10,618,547	98.812	**270,064**	**144,965**	9
*(Velvet+ABySS+SOAP)*							
GAA	2933	429,861	15,592,962	98.896	6,079	6,075	4
MIX	55	700,546	6,089,165	98.554	353,741	380,728	9
Metassembler	194	215,440	5,317,760	97.819	57,802	57,596	3
MAC	42	1,195,331	5,355,147	98.306	**485,219**	**455,989**	**2**

The bolded data indicates the highest value of N50 or NGA50 within each comparison.

**Table 4:** The experimental results of *R. sphaeroides* The highest value of N50 or NGA50 within each comparison.

	Contigs num	Largest contig	Size	Genome fraction	N50	NGA50	MA
Velvet	332	71,713	4,485,514	97.419	23,979	24,300	2
ABySS	382	71,578	4,503,182	97.76	21,441	21,441	1
SOAPdenovo	354	115,051	4,527,360	97.98	33,491	33,491	1
*(Velvet+ABySS)*							
GAA	1745	9,976	8,988,696	98.651	6,650	6,650	3
MIX	274	113,766	4,728,490	97.493	35,067	28,685	35
Metassembler	325	71,713	4,480,778	97.337	23,979	23,979	**2**
MAC	434	126,603	8,043,496	98.718	**53,057**	**52,641**	17
*(Velvet+ABySS+SOAP)*							
GAA	2683	13,133	13,487,438	99.281	7,589	7,571	4
MIX	237	171,915	4,982,251	98.446	51,508	41,915	22
Metassembler	323	71,713	4,477,669	97.269	23,979	23,979	**2**
MAC	122	173,958	4,574,809	98.282	**58,392**	**56,244**	7

The bolded data indicates the highest value of N50 or NGA50 within each comparison.

**Table 5:** The experimental results of *V. cholerae*.

	Contigs num	Largest contig	Size	Genome fraction	N50	NGA50	MA
Velvet	156	246,346	3,944,260	97.563	92,036	63,574	14
ABySS	196	178,118	3,904,784	96.699	61,965	60,272	2
SOAPdenovo	186	246,179	3,924,635	96.94	71,357	65,464	16
*(Velvet+ABySS)*							
GAA	271	170,890	3,958,224	97.207	73,177	56,472	14
MIX	147	310,702	4,038,894	96.915	124,754	91,942	19
Metassembler	150	246,346	3,935,482	97.48	92,036	63,574	**13**
MAC	232	312,914	7,221,147	97.322	**174,216**	**163,176**	21
*(Velvet+ABySS+SOAP)*							
GAA	160	243,299	3,981,614	97.713	110,446	110,446	16
MIX	118	310,703	4,338,139	97.496	112,745	86,841	32
Metassembler	145	246,346	3,914,378	96.972	93,191	63,574	**13**
MAC	87	358,265	3,997,554	97.709	**167,523**	**110,538**	**13**

The bolded data indicates the highest value of N50 or NGA50 within each comparison.

For the case of two assemblies as input, the number of misassemblies of four reconciliation tools have increased somewhat because the quality of input assemblies is relatively low. Even the metrics of N50/NGA50 have remained static or decreased for some tools, such as GAA and Metassembler. By comparison, MAC achieves significant growth in N50 and NGA50 compared to the original input assemblies and the merging results of other reconciliation tools in four datasets, although the number of misassemblies is basically flat.

For the case of three assemblies as input, the metrics of N50/NGA50 of four reconciliation tools have increased in various degrees, due to the addition of high quality assemblies generated by SOAPdenovo, while there is no obvious change in the number of misassemblies for GAA, MIX, and Metassembler. However, MAC not only achieves the obvious increase of N50 and NGA50, but also greatly reduces the number of misassemblies. Especially in the dataset of B.fragilis, the N50 and NAG50 of MAC are 485219 and 455989, which have the growth rate of 79.6% and 214.5%, respectively, compared to the case of two assemblies as input of MAC, and the growth rate of 96.9% and 81.7%, respectively, compared to the high quality input of SOAPdenovo. What’s more, the number of misassemblies has decreased from 9 to 2, which is less than the number of velvet and equals to the number of ABySS.

From the results of **Tables 2**–**5**, MAC outperforms the other reconciliation tools, MAC is not only adapted to merge low quality assemblies to generate a more continuous one, but is also good at fusing different features between assemblies to further improve the contiguity of high quality assemblies, at the same time maintaining the correctness. In addition, we evaluated the computational costs of four tools, as shown in **Table S6** of supplementary material.

## Conclusion

In this study, we have proposed a novel assembly reconciliation tool MAC. MAC classifies all the contigs into “U” and “T” by using alignment results and GC-content of paired-end reads, then identifies consensus blocks between assembly sets, through which unreliable fragments caused by uneven sequencing depth or sequencing errors could be filtered out. In addition, MAC utilizes adjacency algebraic model to fulfill the merging process. The adjacent graph is employed to identify good paths between consensus blocks, which could be used to generate some accurate fusions. Secondly, the classification result of contigs is used to optimize the processing steps of poor paths, through which repetitive regions could be eliminated by splitting contigs and reconstructing the adjacent graph. What’s more, to solve the problem of unknown ranking of input assemblies, MAC designs a scoring function to evaluate the overall quality of assembly sets. The experimental results from four real species of GAGE-B illustrate that MAC performs better than other reconciliation tools.

## Data Availability Statement

The datasets of GAGE-B for this study can be found in https://ccb.jhu.edu/gage_b/. The source code of MAC is available at https://github.com/bioinfomaticsCSU/MAC.

## Author Contributions

LT, ML, F-XW, YP, and JW conceived the original study. LT carried out the analysis of sequencing data and developed the bioinformatics tool. ML contributed to designing the algorithm structure. F-XW, YP, and JW contributed to optimizing the performance of tool. All authors contributed to drafting the manuscript.

## Funding

This work was supported in part by the National Natural Science Foundation of China [No. 61732009, No. 61772557, and No. 61420106009].

## Conflict of Interest

The authors declare that the research was conducted in the absence of any commercial or financial relationships that could be construed as a potential conflict of interest.
