# Supplementary material for: MAC: Merging Assemblies by Using Adjacency Algebraic Model and Classification
Source: Front Genet. 2020 Jan 31;10:1396. doi: 10.3389/fgene.2019.01396 (PMC7005248; doi:10.3389/fgene.2019.01396)
Supplement: Supplementary file 1 [file DataSheet_1.docx]

Supplementary Material

# The pseudo-code of adopting the adjacency algebraic model

| **Algorithm 1. Adopting adjacent algebraic model** |
| --- |
| Input: Two contig sets *O* and *R.* |
| Output: new contig set *M*. |
| 1: Identify all the *consensus blocks (CB)* between *O* and *R*, and mark numbers. |
| 2: Find adjacencies between *CB*. |
| For each *contig(C)* in *O* do |
| if *cb_i_* and *cb_j_* overlap each other with the length of *l* (*l ≤* *l_cmin_** 0.01) then |
| add [*cb_i_, cb_j_*] to adjacency set (*AS_O_).* |
| else add *cb_i_* to *AS_O_*. |
| if the type of *C_i_* is “U” then |
| for each *error position(ep)* in *C_i_* do |
| if *ep_i_* locates at adjacent region then  maintain *ep_i_*. |
| else remove *ep_i_*. |
| else continue. |
| For each *contig(C)* in *R* do |
| if *cb_i_* and *cb_j_* overlap each other with the length of *l* (*l ≤* *l_cmin_** 0.01) then |
| add [*cb_i_, cb_j_*] to adjacency set (*AS_R_).* |
| if the type of *C_i_* is “U” then |
| for each *error position(ep)* in *C_i_* do |
| if *ep_i_* locates at adjacent region then  maintain *ep_i_*. |
| maintain *ep_i_*. |
| else remove *ep_i_*. |
| else continue. |
| 3: Construct the adjacent graph *G =<V, E>* by using *AS_O_* and *AS_R_*. |
| 4: Extract good paths from *G.* |
| 5: Optimize adjacency algebraic model. |
| For each *path(p)* in *G* do  if the type *p* is poor-1 then  if *p* is non-single path then  for each *contig(C_s_)* included in *p* do  if the type of *C_s_* is “U” and exists *ep* then  split *C_s_* at *ep* and repeat step:1-5 for *p.* |
| if the type *p* is poor-1 then |
| if *p* is non-single path then |
| for each *contig(C_s_)* included in *p* do |
| if the type of *C_s_* is “U” and exists *ep* then |
| split *C_s_* at *ep* and repeat step:1-5 for *p* |
| else continue. |
| else continue. |
| else if the type of *p* is poor-2 then |
| if both terminals of the path appear in any good path |
| remove *p*. |
| else maintain *p*. |
| else maintain *p*. |
| when there no good path added, end for. |
| 6: Merge single path into adjacencies. |
| 7: Merge adjacencies to generate *M.* |
| 8: Output *M.* |

# The detail information of the raw reads in GAGE-B

The datasets obtained from GAGE-B (Magoc *et al.*, 2013) are generated by MiSeq technology, including four species: B.cereus, M.abscessus, R.sphaeroides, and V.cholerae, and their statistics are shown in Table S1.

Table S1. The statistics of the raw reads in GAGE-B

|  | B.cereus | M.abscessus | R.sphaeroides | V.cholerae |
| --- | --- | --- | --- | --- |
| Genome size(Mbp) | 5.4 | 5.1 | 4.6 | 4.0 |
| GC content(%) | 35 | 64 | 69 | 48 |
| Read length(bp) | 250 | 250 | 251 | 250 |
| Insert size(bp) | 600 | 335 | 540 | 335 |
| Coverage | 100x | 100x | 100x | 100x |

# The evaluation of contig sets in GAGE-B

The contig sets of GAGE-B are evaluated with Quast (Gurevich *et al*., 2013), the contigs are generated by eight assembly tools, as shown in Tables S2-S5

Table S2. The evaluation of M.abscessus

|  | Number of contigs | Largest contig | MA | NA50 | NGA50 | Genome fraction |
| --- | --- | --- | --- | --- | --- | --- |
| ABySS | 328 | 245660 | 2 | 67488 | 68549 | 99.226 |
| CABOG | 857 | 40668 | 37 | 8294 | 8344 | 96.149 |
| MIRA | 2167 | 279306 | 2265 | 64678 | 74987 | 99.430 |
| MaSuRCA | 326 | 104386 | 62 | 35510 | 37156 | 98.388 |
| SGA | 1230 | 59847 | 225 | 12320 | 12834 | 99.363 |
| SOAPdenovo | 197 | 286460 | 4 | 113272 | 113272 | 99.303 |
| SPAdes | 910 | 498363 | 18 | 209896 | 209896 | 99.414 |
| Velvet | 286 | 226629 | 39 | 40129 | 41485 | 99.053 |

Table S3. The evaluation of B.cereus

|  | Number of contigs | Largest contig | MA | NA50 | NGA50 | Genome fraction |
| --- | --- | --- | --- | --- | --- | --- |
| ABySS | 617 | 430487 | 1 | 130570 | 130570 | 98.963 |
| CABOG | 78 | 258489 | 5 | 150479 | 150479 | 99.142 |
| MIRA | 164 | 311769 | 9 | 100038 | 100038 | 99.066 |
| MaSuRCA | 90 | 766822 | 8 | 246697 | 246697 | 99.165 |
| SGA | 3344 | 106317 | 13 | 22042 | 25512 | 98.943 |
| SOAPdenovo | 123 | 606530 | 0 | 246346 | 246346 | 98.339 |
| SPAdes | 50090 | 346945 | 7 | 103691 | 103691 | 99.110 |
| Velvet | 444 | 91844 | 2 | 24577 | 24465 | 97.787 |

Table S4. The evaluation of R.sphaeroides

|  | Number of contigs | Largest contig | MA | NA50 | NGA50 | Genome fraction |
| --- | --- | --- | --- | --- | --- | --- |
| ABySS | 846 | 71578 | 1 | 21558 | 21441 | 98.730 |
| CABOG | 148 | 127844 | 4 | 41176 | 30459 | 85.519 |
| MIRA | 885 | 67456 | 11 | 15271 | 15445 | 99.172 |
| MaSuRCA | 63 | 241348 | 3 | 142742 | 130714 | 92.037 |
| SGA | 1032 | 44874 | 3 | 9086 | 9108 | 98.875 |
| SOAPdenovo | 441 | 115051 | 0 | 33829 | 33491 | 98.332 |
| SPAdes | 275 | 286217 | 4 | 115059 | 118093 | 99.528 |
| Velvet | 655 | 71713 | 2 | 24248 | 23979 | 98.212 |

Table S5. The evaluation of V.cholerae

|  | Number of contigs | Largest contig | MA | NA50 | NGA50 | Genome fraction |
| --- | --- | --- | --- | --- | --- | --- |
| ABySS | 564 | 178118 | 2 | 60473 | 60278 | 97.559 |
| CABOG | 241 | 140691 | 6 | 33710 | 32784 | 96.853 |
| MIRA | 501 | 450326 | 83 | 106563 | 108689 | 98.324 |
| MaSuRCA | 173 | 255146 | 15 | 76131 | 76131 | 97.671 |
| SGA | 1901 | 105420 | 45 | 23501 | 27303 | 98.272 |
| SOAPdenovo | 312 | 246179 | 4 | 71357 | 68152 | 97.491 |
| SPAdes | 1486 | 741022 | 5 | 246623 | 262160 | 98.632 |
| Velvet | 209 | 246346 | 14 | 67096 | 63574 | 97.723 |

# The evaluation of computational costs

All benchmarks are done on a server with 512G memory and 6 cores (Intel Xeon E5-2620 2.00GHz). Running time and peak memory are shown in Table S6.

Table S6. The evaluation of computational costs

|  | M.abscessus | |  | B.cereus | |  | R.sphaeroides | |  | V.cholerae | |
| --- | --- | --- | --- | --- | --- | --- | --- | --- | --- | --- | --- |
|  | RT | PM |  | RT | PM |  | RT | PM |  | RT | PM |
| *(Velvet+ABySS)* |  |  |  |  |  |  |  |  |  |  |  |
| GAA | 1'20" | 304122 |  | 1'01" | 270012 |  | 2'14" | 359921 |  | 1'09" | 286731 |
| MIX | 4'53" | 542111 |  | 4'34" | 509112 |  | 5'26" | 603513 |  | 4'21" | 478912 |
| Metassembler | 2'20" | 389452 |  | 2'01" | 378105 |  | 3'11" | 419032 |  | 1'46" | 339841 |
| MAC | 1'14" | 293254 |  | 45" | 232033 |  | 1'57" | 319243 |  | 51" | 248112 |
| *(Velvet+ABySS+SOAP)* |  |  |  |  |  |  |  |  |  |  |  |
| GAA | 2'55" | 334511 |  | 1'21" | 298543 |  | 2'58" | 375215 |  | 1'12" | 299051 |
| MIX | 6'03" | 722243 |  | 3'57" | 523950 |  | 5'54" | 618473 |  | 4'55" | 502834 |
| Metassembler | 4'26" | 452093 |  | 2'25" | 403451 |  | 4'34" | 452235 |  | 1'58" | 359823 |
| MAC | 2'04" | 309823 |  | 49" | 278315 |  | 2'13" | 338592 |  | 56" | 261244 |

* Running Time (RT, mins and seconds); Peak Memory (PM, kb)

**References**

Gurevich, A., Saveliev, V., Vyahhi, N., & Tesler, G. (2013). Quast: quality assessment tool for genome assemblies. *Bioinformatics*, 29(8), 1072.

Magoc, T., Pabinger, S., Canzar, S., Liu, X., Su, Q., & Puiu, D., et al. (2013). Gage-b: an evaluation of genome assemblers for bacterial organisms. Bioinformatics, 29(14), 1718-1725.
